# The Ras Superfamily of Small GTPases in Non-neoplastic Cerebral Diseases

**DOI:** 10.3389/fnmol.2019.00121

**Published:** 2019-05-21

**Authors:** Liang Qu, Chao Pan, Shi-Ming He, Bing Lang, Guo-Dong Gao, Xue-Lian Wang, Yuan Wang

**Affiliations:** ^1^Department of Neurosurgery, Tangdu Hospital, Air Force Military Medical University, Xi’an, China; ^2^Beijing Institute of Biotechnology, Beijing, China; ^3^Department of Neurosurgery, Xi’an International Medical Center, Xi’an, China; ^4^The School of Medicine, Medical Sciences and Nutrition, Institute of Medical Sciences, University of Aberdeen, Aberdeen, United Kingdom; ^5^Department of Psychiatry, The Second Xiangya Hospital, Central South University, Changsha, China

**Keywords:** small GTPases, Ras superfamily, Rho subfamily, Rab subfamily, Arf subfamily, Ran subfamily, non-neoplastic cerebral diseases

## Abstract

The small GTPases from the Ras superfamily play crucial roles in basic cellular processes during practically the entire process of neurodevelopment, including neurogenesis, differentiation, gene expression, membrane and protein traffic, vesicular trafficking, and synaptic plasticity. Small GTPases are key signal transducing enzymes that link extracellular cues to the neuronal responses required for the construction of neuronal networks, as well as for synaptic function and plasticity. Different subfamilies of small GTPases have been linked to a number of non-neoplastic cerebral diseases such as Alzheimer’s disease (AD), Parkinson’s disease (PD), intellectual disability, epilepsy, drug addiction, Huntington’s disease (HD), amyotrophic lateral sclerosis (ALS) and a large number of idiopathic cerebral diseases. Here, we attempted to make a clearer illustration of the relationship between Ras superfamily GTPases and non-neoplastic cerebral diseases, as well as their roles in the neural system. In future studies, potential treatments for non-neoplastic cerebral diseases which are based on small GTPase related signaling pathways should be explored further. In this paper, we review all the available literature in support of this possibility.

## Introduction

**FIGURE 1:**
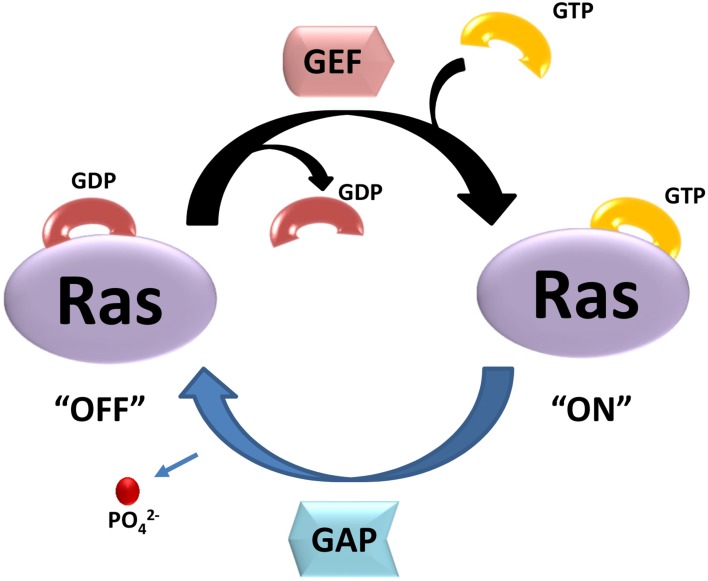
The two state of small GTPases. GTP- and GDP-bound state are regulated by GEFs and GAPs. GEFs stimulate the exchange of GDP for GTP, resulting activation of Ras (“ON”). GAPs promote GTP hydrolysis, and return Ras to GDP-bound state (“OFF”).

Small GTPases are defined by their basic biochemical activity of binding GTP and hydrolyzing it to GDP, which is called the guanosine triphosphate (GTP)/guanosine diphosphate (GDP) cycle (Bourne et al., 1991). Although similar to the heterotrimeric G protein α subunits in biochemistry and function, small GTPases function as monomeric G proteins (Claing, 2013). In addition to their high affinity and hydrolysis activity for GTP, small GTPases have two states, a GTP-bound state and a GDP-bound state. The activated small G-proteins are mainly regulated by three crucial factors: Guanosine nucleotide dissociation inhibitors (GDIs), guanine nucleotide exchange factors (GEFs) and GTPase activator proteins (GAPs) (Buday and Downward, 2008; Vigil et al., 2010; Liu et al., 2017). Figure 1 shows a simple schematic diagram of their function. In this cycle, GAPs promote GTP hydrolysis, and GEFs stimulate the exchange of GDP for GTP (McCormick, 1998). GDIs can be defined as a class of proteins interacted with small GTPases, which not only prevent exchange (maintaining the small GTPases in an off-state), but also prevent the small GTPase from localizing at the membrane (Cherfils and Zeghouf, 2013). Importantly, these molecular switches affect almost all cellular processes such as gene expression, microtubule organization, cytoskeleton reorganization, and vesicular and nuclear transport (Johnson and Chen, 2012). Small GTPases can also be influenced by post-translational modifications such as phosphorylation or ubiquitination, which can regulate protein stability and subcellular localization (Ahearn et al., 2011).

**FIGURE 2:**
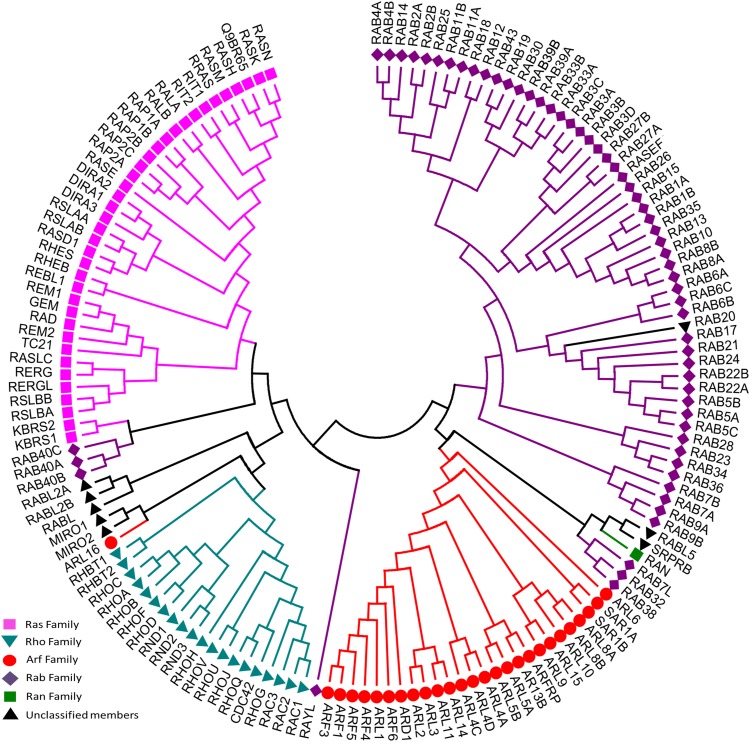
Evolutionary relationships of human Ras superfamily. The evolutionary history was inferred using the Neighbor-Joining method. After removing identical sequences, 162 amino acid sequences (Supplementary Table S1) are used for the analysis. All ambiguous positions were removed for each sequence pair. The original classification was indicated by different colors: Ras (lilac), Rho (cyan), Rab (purple), Arf (red), Ran (green), and Unclassified members (blank). Evolutionary analyses were conducted in MEGA7.

To date, 167 small GTPases have been identified in humans (Supplementary Table S1) (Rojas et al., 2012; Liu et al., 2017). We compared the G-domin region of those small GTPases, and found that there exist 5 repeated sequences. After removing 5 repeated amino acid sequences that are identical, the resulting 162 amino acid sequences were analyzed and a sequence-homology based phylogenetic tree (Figure 2) was constructed using the Neighbor-Joining method (Saitou and Nei, 1987). According to sequence and functional similarity, small GTPases can be divided into five main families whose eponymous members are Ras, Rho, Rab, ADP-ribosylation factor (Arf) and Ran (Wennerberg et al., 2005). Ras family members, the first members of the superfamily to be discovered, are signal nodes mediating the responses to various extracellular stimuli and can regulate cell proliferation, differentiation, morphology, and apoptosis by binding to a variety of effector molecules with different catalytic activities to regulate the cytoplasmic signaling network (Karnoub and Weinberg, 2008). The Rho family includes more than 20 proteins. This family is known for its role in actin cytoskeleton remodeling and cell polarity (Fransson et al., 2003; Heo and Meyer, 2003). The three best known members are Rho (A, B, C), Rac and Cdc42 (Takai et al., 2001; Shenoy and Lefkowitz, 2011; Reiter et al., 2012; Claing, 2013). Among them, RhoA, Rac1, and Cdc42 have been studied the most (Schwartz and Shattil, 2000; Rolfe et al., 2005; Grosshans et al., 2006). The Rab family is by far the largest family of the Ras superfamily in humans (Rojas et al., 2012). Proteins of this family participate in vesicle formation, movement and fusion, and vesicular cargo trafficking (Pereira-Leal and Seabra, 2001; Segev, 2001). The Arf family includes 30 proteins and is also involved in vesicle trafficking. Ran is the only member found in the Ran family, which is present in all eukaryotic lineages and involved in nuclear transport (Weis, 2003).

**FIGURE 3:**
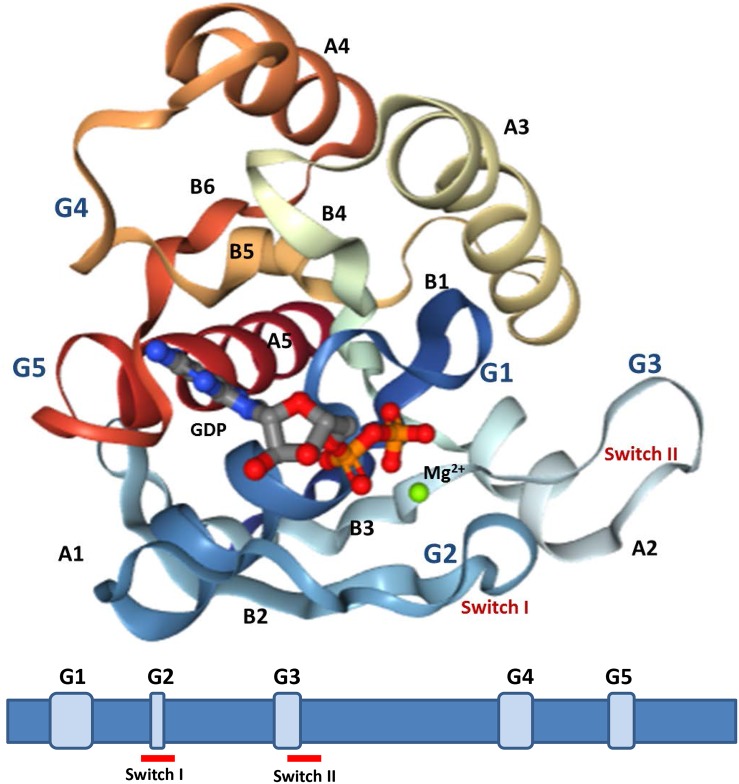
Structures analysis of Ras. The crystal structures of Ras GDP Mg^2+^ complex (PDB 4q21) is showed (upper). This structure contains five α-helices (A1–A5), six β-strands (B1–B6), and five polypeptide loops (G1–G5) and the position relationship among various parts is displayed (below).

**FIGURE 4:**
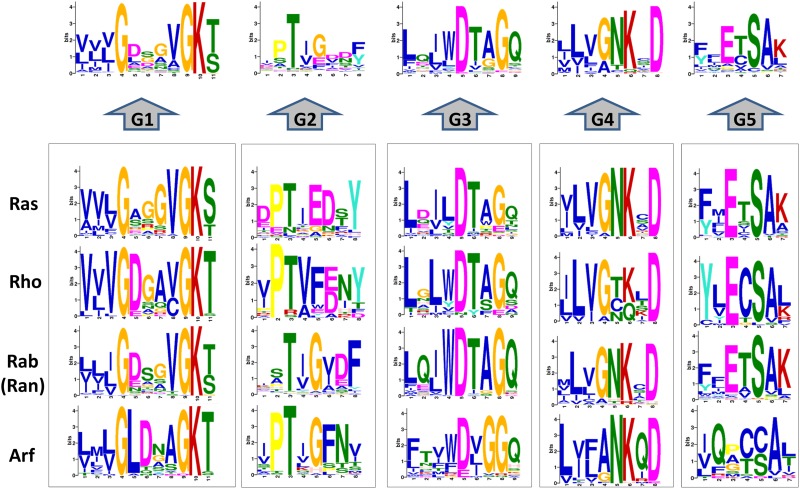
Conservation analysis of the 167 human G-domain of Ras superfamily and its subfamilies (http://meme-suite.org/). Considering that the Ran only has one sequence and is a branch of Rab subfamily, we incorporate Ran into Rab family.

All the proteins have a GTP-binding domain of about 20 kDa, also known as the G-domain (Bourne et al., 1991; Takai et al., 2001). As shown in Figure 3, this domain consists of five α helices (A1–A5), six β-strands (B1–B6) and five polypeptide loops (G1–G5). This domain is highly conserved overall, and especially the loops (Paduch et al., 2001). When bound to GDP, small GTPases are inactive. The two states have similar conformations and can be distinguished by two functional loop regions: switch I (corresponds to the G2 loop) and switch II (corresponds to the G3 loop and part of the A2 helix) (Figure 3) (Jurnak, 1985; Paduch et al., 2001). The G1 loop is located between the B1 strands and A1 helix with the motif X_1_X_1_X_1_GXXXXGK (S/T), where X_1_ is leucine (L), valine (V) or isoleucine (I), and X is any amino acid. The α- and β-phosphate groups can be bound to this motif (Knihtila et al., 2015). The G2 loop, with only one conserved threonine (T), that connects the A1 helix and the B2 strand is responsible for the binding of Mg^2+^ via conserved amino acid residues. The G3 loop is at the N-terminus of the A2 helix with the motif XXXXDXXGX. Its main function is to bind Mg^2+^ and the γ-phosphoric acid group of GTP or GDP. The G4 loop containing the motif XXX (G/A) (T/N) KXD and the G5 loop are mainly responsible for the recognition of the guanine base (Figure 4). Although the G-domain is conserved in the superfamily, there are also individual difference among the different small GTPase families. For example, in G1 loop, the sixth position G is only conserved in the Rho, Rab, and Arf families. On the other hand, covalent post-translational modification by lipids is another feature of small GTPases. These modifications are essential for facilitating membrane association and subcellular localization, which is critical for their respective biological activities (Hancock, 2003; Prior and Hancock, 2012). The C-terminal cysteine residue of the motif can be prenylated by farnesyltransferase or geranylgeranyltransferase. This modification was observed for most Ras, Rho, and Rab family members despite the different motifs, which is composed of CAAX (C is Cys, A is aliphatic and X is any amino acid) for Ras and Rho family proteins, and CC, CXC, CCX, CCXX, or CCXXX for the Rab family (Cox and Der, 2002; Wennerberg et al., 2005). Some Ras superfamily members without lipid modification, such as Rit, RhoBTB, Miro, and Sar1, can also associate with membranes (Wennerberg et al., 2005). Arf family proteins lack C-terminal lipid modification signals and some members are modified at their N-termini by a myristoylation (Colicelli, 2004). By contrast, Ran does not exhibit any detectable modification by lipids and binding to membranes (Wennerberg et al., 2005).

Studies of the functions of Ras superfamily proteins in modulating the diverse signaling cascades have established many current fundamental paradigms of signal transduction, mainly based on decades of oncological investigations. The awareness of aberrant Ras-related signaling in the pathogenesis of other human disorders has also risen in recent years. Here we highlight the critical roles of each family of the Ras superfamily in non-neoplastic cerebral diseases (Tables 1–3), which in turn may suggest novel therapeutic targets for developmental diseases and cognitive impairments.

**Table 1:** Gene mutations related to Small GTPases (Ras family).

Gene mutations	Related disease	Proteins	Pathway/mechanism	References
*PTPN11*	NS	SHIP2: Protein tyrosine phosphatase	Increased RAS/MAPK	Siegfried et al., 2017
*PTPN11*	NS-ML	SHIP2: Protein tyrosine phosphatase	Increased AKT/mTOR	Tajan et al., 2015
*SOS1*	NS	Son of sevenless homolog 1	Increased RAS/MAPK, Rac, and Stat3	Van Trier et al., 2017
*RAF1*	NS	v-Raf-1 murine leukemia viral oncogene homolog 1	Increased RAS/MAPK	Yin et al., 2017
*RAF1*	NS-ML	v-Raf-1 murine leukemia viral oncogene homolog 1		
*KRAS*	NS	V-Ki-Ras2 Kirsten rat sarcoma viral oncogene homolog	Increased RAS/MAPK	Tartaglia et al., 2011
*KRAS*	CFCS			
*NRAS*	NS	Neuroblastoma Ras viral (V-Ras) oncogene homolog	Increased RAS/MAPK	Ekvall et al., 2015
*SHOC2*	NS	soc-2 suppressor of clear homolog	SHOC2-MRAS-PP1 complex positively regulates RAF activity	Young et al., 2018
*SHOC2*	NS-LAH	soc-2 suppressor of clear homolog		
*BRAF*	NS	Serine/Threonine-Protein Kinase B-Raf	Increased RAS/MAPK	Tartaglia et al., 2011
*BRAF*	CFCS	Serine/Threonine-Protein Kinase B-Raf	Increased RAS/MAPK, Decreased p38 and AKT	Tartaglia et al., 2011
*RIT1*	NS	Ras-Like Without CAAX 1	Increased RAS/MAPK	Aoki et al., 2013
*RRAS*	NS	Related Ras viral (R-Ras) Oncogene Homolog	Related RAS Viral (R-Ras) Oncogene Homolog; Increased RAS/MAPK	Flex et al., 2014
*MAP3K8*	NS	RAS/MARK pathway	Increased RAS/MAPK	Tidyman and Rauen, 2016a
*MEK1*	CFCS	MEK 1: Mitogen-activated protein kinase kinase 1	Increased RAS/MAPK	Dentici et al., 2009
*NF1*	NF-1	Neurofibromin	PI3K/mTOR/AKT pathway	Arun et al., 2013
*SPRED1*	LS	Sprouty-related EVH1 domain containing protein 1	Increased RAS/MAPK and JAK2	Hirata et al., 2016
*SPRED2*	OCD	Sprouty-related EVH1 domain containing protein 2	Loss of SPRED2	Ullrich et al., 2018
*HRAS*	CS	Harvey rat sarcoma viral oncogene homolog	Increased RAS/MAPK	Bertola et al., 2017
*RIT2* (rs12456492)	Parkinson’s disease	CD33	rs12456492 polymorphism is associated with increased CD33 expression	Liu et al., 2015
*RIT2* (rs12456492)	Essential tremor	–	–	Emamalizadeh et al., 2017
*RIT2* (rs16976358)	Autism spectrum disorder	Regulatory motif of the SOX transcription factor	rs16976358 variant	Hamedani et al., 2017
*RIT2* (rs16976358)	Schizophrenia	–	CNV	Rees et al., 2014; Tansey et al., 2016
*RIT2* (rs16976358)	Bipolar disorder	–	–	Emamalizadeh et al., 2017
*RIT2* (rs4130047)	Autism spectrum disorder	–	–	Hamedani et al., 2017


**NS, Noonan syndrome; NS-ML, Noonan syndrome with multiple lentigines; NS-LAH, Noonan-like syndrome disorder with loose anagen hair; CS, Costello syndrome; CFCS, Cranio-facio-cutaneous syndrome; LS, Legius syndrome; OCD, Obsessive compulsive disorder; NF-1, Type 1 neurofibromatosis; CNVs, Copy number variations*.*

**Table 2:** Interacting proteins related to Small GTPases (Ras family).

Small GTPase	Interacting proteins	Related diseases/function	Pathway/mechanism	References
Rit2 (Rin)	NGF	Alzheimer’s disease	Increased RAS/MAPK	Besga et al., 2017
		Huntington’s disease	Increased AKT/mTOR	Chaldakov et al., 2009; Zhang et al., 2013
	RIT1	Noonan syndrome	Increased RAS/MAPK, Rac, and Stat3	Yaoita et al., 2016
		Mental retardation, microcephaly, and epilepsy		Sonmez et al., 2017
	TECR	Non-syndromic mental retardation	Increased RAS/MAPK	Caliskan et al., 2011
	NTRK1	Hereditary sensory and autonomic neuropathy type V	A mutation in the *NTRK1*/high-affinity nerve growth factor (TrkA) gene	Houlden et al., 2001
		Congenital insensitivity to pain with anhidrosis		
	POU4F1	Autism	Copy number variations (CNVs)	Indo, 2001
Rit1 (Rit)	B-Raf	Neuronal development and regeneration	Activation of the B-Raf/ERK and p38 MAP kinase cascades	Salyakina et al., 2011
	p38 MAPK	Neurite outgrowth and cell survival		
	MK2	Cell survival	Rit GTPase-p38-MK2-AKT 9 kinase survival pathway	Shi and Andres, 2005
	HSP27	Cell survival	MK2 signaling complex	Shi et al., 2013
	Akt	Cell survival	p38–MK2–HSP27-Akt activation	Shi et al., 2011
	RGL3	RalA activation	A candidate effector for Rit and Ras	Shao and Andres, 2000
Rheb	mTOR	Promoting growth, cell cycle progression and inhibition of autophagy	Growth factor-induced mTORC1 activation	Sokolov et al., 2018
	TSC complex	Form a complex with Rheb at the lysosomal membranes	Growth factor-induced mTORC1 activation	Lim and Crino, 2013
	PLD1	Rheb binds and activates phospholipase D1 (PLD1) in a GTP-dependent manner	mTORC1 activation	Zheng et al., 2014
	GAPDH	GAPDH regulates mTOR activity by sequestering the Rheb.	Rheb–GAPDH interaction	Lee et al., 2009
	FKBP38	Coordinate membrane targeting of Rheb	Rheb interacts with FKBP38 and prevents its association with mTOR	De Cicco et al., 2018
	RASSF1	Rheb form complex with RASSF1A to coordinate Hippo and TOR signaling	Hippo pathway activator	Nelson and Clark, 2016
	NIX LC3	Rheb interacts with mitochondrial autophagic receptor Nix and the autophagosomal protein LC3-II.	Activation of mitophagy	Melser et al., 2013 Sugiura et al., 2015

	Syntenin	PDZ protein syntenin preferentially binds to the GDP-bound form of Rheb.	Rheb-syntenin signaling	Sato et al., 2015
	CAD	Rheb binds to CAD protein, a multifunctional enzyme required for the synthesis of pyrimidine nucleotides.	CAD binding is more pronounced with Rheb2 than with Rheb1	Tyagi et al., 2015
	PERK	Rheb inhibits protein synthesis by activating the PERK-eIF2α signaling	Phosphorylation of eIF2α and PERK interplay	Shahani et al., 2017
	BACE1	Aging and Alzheimer’s disease. Forebrain Rheb promotes aging-associated cognitive defects	Rheb depletion increased the levels of BACE1	


**Table 3:** Small GTPases and Related Diseases (Rho, Arf, Rab, and Ran families).

Small GTPase	Related disease	Pathway/mechanism	References
Rac3	A novel neurodevelopmental syndrome	De novo monoallelic missense variants in Rac3, including one recurrent change	Costain et al., 2019


RhoA	Diabetic Parkinson’s disease or dementia	Accelerated neuron loss via the 5-hydroxytryptamine 2 receptor	Zimering, 2018


Cdc42	Epileptic-seizures	Regulating synaptic inhibition	Zhang Y. et al., 2015


Rac1	Fragile X syndrome	Rac1 is necessary for normal spine development and long-term synaptic plasticity	Bongmba et al., 2011


Rac1	Epilepsy	Patients suffering from temporal lobe epilepsy (TLE) and experimental epileptic rats	Li et al., 2016


RhoB	Glioblastoma	Cytokine-induced STAT3 activation, activated p53 and p21	Ma et al., 2015


Rac1 and Cdc42	Developmental delay, secondary macrocephaly, seizures, and ataxic gait	De novo PAK1 mutations c.392A>G (p.Tyr131Cys) and c.1286A>G (p.Tyr429Cys)	Harms et al., 2018


Rac1	Drug withdrawal	Rac1-dependent GABAAR endocytosis, synaptic plasticity as well as learning and memory	Wang et al., 2017


Rac1	Alcohol use disorders	Rac1/Arfaptin/Arf6 pathway	Peru Y Colón de Portugal et al., 2012


RhoA	Cocaine	Phosphorylated ERM levels and synaptic changes in the NAcc	Kim et al., 2009


Rab7	Parkinson’s disease	Clearance of α-synuclein aggregates	Saridaki et al., 2018


Rab11	Parkinson’s disease	Reduces α-synuclein aggregation and toxicity	Chutna et al., 2014


Rab39b	Parkinson’s disease	Reduce steady-state levels of α-synuclein	Wilson et al., 2014


Rab	Infantile encephalopathy	Rab GTPase-Activating Protein dysregulates mTOR signaling	Chong et al., 2016


Rab1A	Alzheimer’s disease	Mediates Golgi dynamics	Mohamed et al., 2017


Rab3A	Alzheimer’s disease	Localizes in presynaptic vesicles and regulates exocytosis	Udayar et al., 2013


Rab4	Alzheimer’s disease	Regulates endosomal recycling	Ginsberg et al., 2011


Rab5	Alzheimer’s disease	Modulates endosomal membrane trafficking, sorting and endosomal fusion	Ginsberg et al., 2010a,b


Rab6	Alzheimer’s disease	Regulates retrograde Golgi-ER trafficking	Scheper et al., 2007


Rab7A	Alzheimer’s disease	Controls transport to late endosomes and lysosomes/regulates tau secretion	Ginsberg et al., 2011; Rodriguez et al., 2017; Zafar et al., 2017


Rab8	Alzheimer’s disease	Modulates polarized trafficking	Shimohama et al., 1999; Kametani et al., 2004; Li et al., 2012


Rab10	Alzheimer’s disease	pRab10-T73, decrease in Aβ42 and Aβ42/Aβ40 ratio	Ridge et al., 2017; Yan et al., 2018


Rab11A/Rab11B	Alzheimer’s disease	Regulates both endocytic and exocytic trafficking pathways	Udayar et al., 2013; Buggia-Prevot et al., 2014; Xu et al., 2015; Zhao et al., 2015


Rab14	Alzheimer’s disease	Endocytic recycling and Golgi-endosome trafficking	Linford et al., 2012; Prekeris, 2012


Rab17	Alzheimer’s disease	Involved in phagocytic removal of apoptotic cells	Udayar et al., 2013


Rab24	Alzheimer’s disease	Involved in autophagy	Ginsberg et al., 2010a


Rab27	Alzheimer’s disease	Regulates exocytosis, endocytosis and phagocytosis	Ginsberg et al., 2011


Rab36	Alzheimer’s disease	Involved in late endosome and lysosome distribution	Udayar et al., 2013


Rab3a	Glioma	Increases cyclin D1 expression	Kim et al., 2014


Rab38	Glioblastomas	Whole genome mRNA expression microarray data on 220 glioma samples from the Chinese glioma genome atlas	Wang and Jiang, 2013


Rab28	Cone-rod dystrophy	Retinal dystrophies	Roosing et al., 2013


Arf1	Creutzfeldt-Jakob disease	Arf/Rho/MLC signaling	Zafar et al., 2015


Arl2 and Arl3	Retina disease	Participate in the trafficking of lipidated membrane-associated proteins and colocalize in the inner segment with UNC119A and PDEδ	Veltel et al., 2008a; Zhang H. et al., 2015; Hanke-Gogokhia et al., 2016a,b; Moshiri et al., 2017


Arf6	Alcohol use disorders	Rac1/Arfaptin/Arf6 pathway	Peru Y Colón de Portugal et al., 2012


Arl6	Diabetic retinopathy	Regulates VEGFR2 trafficking and signal transduction	Zhu et al., 2017


Arl6	ALS	Potential driver pathophysiological events involving endoplasmic reticulum stress and autophagy	Zhai et al., 2015


Arf72A	Retina disease	Rescues the ninaE(D1)-related membrane accumulation and suppresses ninaE(D1)-triggered retinal degeneration	Lee et al., 2011


Arl2	Bardet–Biedl syndrome type 3	Disrupt a threonine residue important for GTP binding and function of several related small GTP-binding proteins	Fan et al., 2004


Ran	Frontotemporal dementia	Regulates nuclear import via TDP-43 pathway	Ward et al., 2014


Ran	Alzheimer’s disease	Transcription regulators in the nucleus	Mastroeni et al., 2013


Ran	Glioblastoma multiforme	The Survivin-Ran complex	Guvenc et al., 2013




*ALS, amyotrophic lateral sclerosis.*

## The Ras Family in Non-Neoplastic Cerebral Diseases

### The Ras Family

As the first described and prototypical members of the small GTPase superfamily, Ras family proteins are almost universal components of signaling pathways in eukaryotic organisms, including vertebrates, invertebrates, and yeasts (Goitre et al., 2014). Ras genes were first identified as oncogenes, and were named after the Rat Sarcoma gene in the 1970s during the extensive study of acutely transforming retroviruses isolated from mice, rats, and other animals (Cox and Der, 2010). Following years of investigation, the widespread prevalence of Ras mutations in the context of carcinogenesis has now been widely recognized. This has inspired multiple attempts to find Ras inhibitors. However, as for the role of the Ras family in non-neoplastic diseases, we are still at the beginning stage of discovering how the related proteins and their precise mechanisms control and modulate non-oncogenic processes (Johnson and Chen, 2012).

According to their evolutionarily conservation in structural, biochemical, and functional levels, the Ras family is composed of over 36 members, with six major subfamilies: Ras subfamily (H-Ras, K-Ras, N-Ras, etc.), Ral (RalA, RalB), Rap (Rap1, Rap2), Rad (Rad, Gem, etc.), Rheb (Rheb1, and Rheb2 or RhebL1) and Rit (Rit1 or Rit, Rit2 or Rin, and *Drosophila dRic*) (Goitre et al., 2014).

Most Ras family proteins are predominantly localized on the intracellular surface of the plasma membrane as a result of C-terminal prenylation (Colicelli, 2004). They can be activated in response to multiple extracellular stimuli in the form of growth factors and small molecules, which thereby transduce signals to the intracellular environment to regulate cytoplasmic signaling networks (Ye and Carew, 2010).

The best-characterized downstream signaling cascade of Ras family proteins is the Mitogen-Activated Protein Kinase (MAPK) cascade, mainly the Extracellular signal-Regulated Kinase 1/2 (ERK) of the MAPK family (Ye and Carew, 2010). Ras family proteins can directly bind to the regulatory domain of Raf (c-Raf or b-Raf) and subsequently phosphorylate and dephosphorylate it at multiple sites for full activation. In turn, activated Raf phosphorylates and activates MAP Kinase or ERK Kinase (MEK), and finally targets transcription factors to induce gene expression (Lange-Carter and Johnson, 1994). Take Rap subfamily members for example. Rap1 can activate ERK by activating b-Raf (expressed predominantly in the brain) and p38MAPK, which implies a pivotal role in synaptic depression (Grewal et al., 2000; Kanda and Watanabe, 2007). Furthermore, Rap2 is involved in the activation of c-Jun N-terminal Kinase (JNK) (Machida et al., 2004). In addition to the Raf–MEK–ERK cascade, other well-known pathways include the phosphatidylinositol 3-kinase (PI3K), RalGEF-Ral, and phospholipase C epsilon (PLCε) pathways (Pulciani et al., 1982; Santos et al., 1984).

The Rit subfamily is composed of two classes, Rit and Rin (Ras like without CAAX2, Rit2), each containing a well-conserved GTPase core (G1–G5). Distinct patterns of developmental expression for both proteins were found, whereby Rit is widely expressed throughout development, while Rin shows delayed expressed limited to later stages of embryonic neuronal development (E14), with highest Rin expression found in the adult brain (Shi et al., 2013; Hamedani et al., 2017). Activated Rit was identified as playing a critical role in the regulation of neuronal morphogenesis, especially enhancing axonal growth, but with the opposite effect on dendritic growth (Lein et al., 2007).

Rin is involved in calcium-mediated processes in cells and binds to calmodulin through a C-terminal binding motif, regulating the Rin-mediated pathway (Hoshino and Nakamura, 2002; Daneshmandpour et al., 2018). Rin has an important role in intracellular signaling and interacts with DAT (dopamine active transporter) in lipid raft microdomains (Navaroli et al., 2011).

The Rheb subfamily (Ras homolog protein enriched in the brain) is a member of the Ras family that is highly conserved among different organisms (Urano et al., 2001; Schoneborn et al., 2018). Two different Rheb genes have been identified in mammalians, *RHEB1* (from here on *REHB*), and *RHEB2* (also as *RHEBL1*). RHEB is expressed in various human tissues, but RHEB2 is mainly expressed in the brain tissue, such as cerebral cortex, occipital pole, frontal, and temporal lobes (Saito et al., 2005; Schoneborn et al., 2018).

Mammalian target of rapamycin (mTOR) is a serine-threonine kinase that integrates signals to regulate cell growth and metabolism. In the brain, Rheb functions as a key activator of mTORC1, and deletion of RHEB causes decreased cortical thickness and defective myelination (Lee et al., 2015).

RalA and RalB are monomeric GTPases belonging to the Ral subclass, and both are expressed in the nervous system (Teodoro et al., 2013). Ral can be activated either by Ras indirectly via a Ral-GEF (Guanine nucleotide Exchange Factor) or by Ca^2+^/calmodulin binding, and it is inactivated by PKC phosphorylation of the effector Sec5 (Harvey et al., 2008; Chen et al., 2011). During brain development, it is considered that Ral is involved in the asymmetric division of neuroblasts, neuronal migration in the neocortex, neurite branching, and activity-dependent spine growth (Das et al., 2014). RalA regulates axon initiation in cortical neurons by promoting an interaction between the exocyst protein complex, which is one of its major effectors, and the Par3–Par6–aPKC complex, an evolutionarily conserved master regulator of cell polarity. However, in other cell migration process, such as tumor metastasis, it seems that RalB may play a more important role than RalA (Zago et al., 2017).

The Rap subfamily is composed of five related proteins, Rap1 (A and B) and Rap2 (A, B, and C), which have overlapping functions and expression patterns (Nakamura et al., 2013; Zhang L. et al., 2018). Similar to other members of the Ras family, Rap proteins work as molecular switches of multiple signal transduction cascades and are linked to several genetic defects related to mental, neurological and psychiatric disorders (Volk et al., 2015). Rap1 has been intensively investigated for its function in integrin-mediated cell adhesion and the regulation of cell–cell junction integrity (Jossin, 2011). A recent study indicates that Rap1 and Rap2 predominantly signal synaptic depression via the lysosomal p38MAPK and the bulk membrane JNK pathway, respectively, regulating different forms of synaptic plasticity (Zhang L. et al., 2018). Especially, Rap1B acts as a core molecule in the signaling network responsible for neuronal polarization (Nakamura et al., 2013).

### Diseases Related to the Ras Family

Owing to the Ras family proteins’ essential role in modulating a wide range of cellular processes, several human diseases are caused by the dysregulation or dysfunction of related signaling pathways. These include cancer, developmental-, neurocognitive- and neurodegenerative disorders, as well as metabolic and cardiovascular diseases (Nakhaei-Rad et al., 2018). In this section, we reviewed some Ras family related developmental disorders and neurological diseases.

#### RASopathies

The RASopathies are the largest known group of rare human developmental disorders that are caused by germline mutations in genes that encode proteins of the RAS/MAPK pathway, resulting in its hyperactivation (Tajan et al., 2018). As mentioned above, the RAS/MAPK pathway is essential for the development of mammalian tissues, controlling a variety of cellular activities, such as cell cycle and growth, differentiation, metabolism and senescence (Slack, 2017). Although this signaling pathway is often summarized as a well-established cascade, there are still many gray areas and multilevel regulations, such as transcriptional control, posttranslational modifications, protein-protein interactions, and crosstalk with other signaling pathways. In this section, we mainly focus on individual symptoms, phenotypes and most evidenced gene mutations to the RASopathies. Further detailed information on comprehensive functional and pathophysiological consequences could be referred to suggested reviews (Atay and Skotheim, 2017; Simanshu et al., 2017).

The RASopathies includes neurofibromatosis type 1 (NF1), Legius syndrome (LS), capillary malformation–arterio-venous malformation syndrome (CM-AVM), Costello syndrome (CS), cardio-facio-cutaneous syndrome (CFC), Noonan syndrome (NS) and Noonan syndrome with multiple lentigines (NS-ML) (Simanshu et al., 2017; Dard et al., 2018). Although RASopathies exhibit unique phenotypes, they share many overlapping clinical characteristics due to the common pathway dysregulation, such as partial facial anomalies, cognitive impairment, and congenital heart defects (Nakhaei-Rad et al., 2018). As a whole, the RASopathies affect approximately 1 per 1,000 live births (Tajan et al., 2018). The initial diagnosis of a RASopathy patient is based on the clinical recognition of phenotypic features, and the clinical diagnosis is then confirmed by molecular genetic tests. To date, with the accumulation of genotype and phenotype correlation data, there are more than 20 gene mutations has been associated with RASopathies. Most mutations causing RASopathies occur at conserved positions within the RAS/MAPK pathway, providing a genetic foundation for their diagnosis and pathophysiology (Simanshu et al., 2017). Table 1 has listed several gene mutations related to small GTPases, especially the Ras family and RASopathies. However, considering the heterogeneity of a given syndrome and the pleiotropic roles during development and homeostasis maintenance, it is still difficult and fragmental to classify the causal genes and mutations involved with the genotype/phenotype correlations based on current progress.

NS was first described by Jacqueline Noonan about 50 years ago, and is also the most frequent RASopathy. Its estimated prevalence is between 1 in 2,000–2,500 newborns (Noonan, 1968; Tajan et al., 2018). It is an autosomal dominant disorder and the most genetically diverse RASopathy, which is clinically characterized by facial dysmorphic features, congenital heart defects, and growth retardation. More than half of patients with NS are identified as having mild to moderate developmental delay/learning disability, notably with social and communication difficulties, attention deficit, and language impairment (Cesarini et al., 2009). Most of the mutations responsible for NS affect familiar components of Ras pathways. The protein tyrosine phosphatase non-receptor type (*PTPN11*), encoding SHP2, is the first and major NS disease gene, which was found mutated in 50–60% of patients with NS (Atay and Skotheim, 2017). Other less common or rare mutation genes are related to the RAS/MAPK pathway as well, including SOS1, SOS2, H-Ras, K-Ras, R-Ras, N-Ras, M-Ras and Rit1, the GAP RasA2, the kinases CRAF, BRAF, MEK1, MEK2, and MAP3K8, CBL, MYST4, A2ML1, SHOC2, and its binding partner PP1 (Tidyman and Rauen, 2016b). SHOC2 mutations have been reported in combination with mutations in PTPN11 (SHP2) in NS (Ekvall et al., 2011). The diversity of the affected genes has determined the NS is the most genetically heterogeneous RASopathy. However, the role of these proteins in regulating the RAS/MAPK pathway is still not clear, and the underlying functional mechanisms and genotype/phenotype correlations remain to be elucidated (Tidyman and Rauen, 2016b; Simanshu et al., 2017).

NS-ML, formerly known as LEOPARD syndrome, is an autosomal dominant disorder that has a prevalence of fewer than 1 in 100,000 newborns (Sarkozy et al., 2008). However, considering that the phenotype of NS-ML is very close to that of NS, the diagnosis can be more difficult (Sarkozy et al., 2008). Distinctive clinical features for NS-ML are a high prevalence of hearing deficits (20%) and multiple pigmented skin lesions called lentigines, mostly starting at school age (90%) (Sarkozy et al., 2008). Missense mutations in PTPN11 (Legius et al., 1993; Digilio et al., 2006) and rarely in RAF1 (Pandit et al., 2007) and BRAF (Digilio et al., 2006) are associated with NSML. Loss of function mutations in the PTPN11 gene result in reduced SHP2 activity, which is not found in NS (Digilio et al., 2002; Kontaridis et al., 2006). A recent study implied that the Ras-related Akt-mTOR signaling pathway is implicated in NS-ML phenotypes (Marin et al., 2011).

Noonan-like syndrome with loose anagen hair (NSLAH), also known as Mazzanti syndrome, is phenotypically close to NS, but patients display distinctive hyperactive behavior and pathognomonic hair anomalies (Gripp et al., 2016). SHOC2 and PPP1CB have been indicated with the onset of the disease (Atay and Skotheim, 2017). NF1, the second-most frequent RASopathy, is an autosomal dominant genetic disorder that was first described in 1882 (Garcia-Romero et al., 2016; Simanshu et al., 2017). The incidence of NF1 is 1 per 2,500–3,000 in newborns, with approximately 50% of NF1 patients inheriting the mutation from a parent (Williams et al., 2009). The characteristic feature used for NF1 diagnosis is the presence of café-au-lait macules. Benign tumors (neurofibromas and optic pathway gliomas), iris Lisch nodules, bone malformations (limb pseudarthrosis), cardiac malformations, brain malformations, seizures, and mild neurocognitive impairment can aid the diagnosis (Huffmeier et al., 2006; Stevenson et al., 2006). NF1 is caused by mutations in the NF1 gene on chromosome 17q11.2, which encodes neurofibromin, a GAP that negatively regulates Ras (Cawthon et al., 1990; Legius et al., 1993). NF1 mutations result in the loss of function of neurofibromin, which in turn reduces Ras GTPase activity and finally increases the levels of active GTP-bound Ras. Studies have suggested that neurofibromin can act on M-Ras, R-Ras, and R-Ras2 (a.k.a. TC21) (Ohba et al., 2000). R-Ras and N-Ras activate PI3Kγ, while M-Ras recruits SHOC2/PP1c to the plasma membrane to regulate SCRIB activity (Rodriguez-Viciana et al., 2004; Young et al., 2013). As a consequence, the loss of neurofibromin may have broader impacts on cells than the activation of H-Ras, N-Ras, and K-Ras proteins themselves (Simanshu et al., 2017).

Legius syndrome is a milder form of neurofibromatosis type 1, which shares phenotypic features with NF1, albeit in less severe form (Tajan et al., 2018). However, the NF1 gene is intact and heterozygous inactivating mutations in the SPRED1 gene on chromosome 15q13.2 occur in LS (Brems et al., 2007). SPRED1 functions as a negative regulator of Ras by inhibiting the phosphorylation of Raf (Wakioka et al., 2001). The SPRED1 proteins are essential for the interaction of neurofibromin with Ras at the plasma membrane. It is suggested that SPRED2 and SPRED3 proteins can partially compensate the loss of SPRED1 function (Simanshu et al., 2017). Although SPRED1 binding does not affect neurofibromin’s GAP activity, it nevertheless plays an important role in enabling neurofibromin to downregulate Ras activity at the plasma membrane.

CFCS is a rare autosomal dominant disease with multiple congenital anomalies that affects 1/800,000 newborns (Pierpont et al., 2014). It shares many overlapping features with NS (e.g., heart defects, short stature, and facial features) but is additionally characterized by thick scaly skin, delayed growth and cardiac malformations (Rauen, 2007). Affected individuals frequently have severe neurological disturbances and mental retardation (Yoon et al., 2007). CFCS is caused by heterozygous mutations in BRAF (75% of cases), and less frequently in the MAP2K1 (MEK1), MAP2K2 (MEK2) (20% of cases) and *k-ras* genes (Rodriguez-Viciana et al., 2006). BRAF is a downstream effector of Ras, and activating BRAF can increase the activation of the MAPK pathway by CRAF (Heidorn et al., 2010).

Costello syndrome is a multiple congenital anomaly syndrome caused by heterozygous activating germline mutations in HRAS. A common and distinctive feature of CS among RASopathies is an increased risk of developing cancers such as rhabdomyosarcomas and neuroblastomas. A recent study has found an increased energy expenditure (EE) in patients with CS, resulting in growth failure (Leoni et al., 2016).

CM-AVM is associated with arteriovenous malformations and fistulas, and is caused by heterozygous inactivating mutations in RASA1 (Eerola et al., 2003). RASA1 encodes p120-RasGAP, which is a negative regulator of the RAS/MAPK signal transduction pathway. RASA1 mutations have also been associated with the related condition known as Parkes Weber syndrome (Banzic et al., 2017).

From the functional perspective, the global relationship for different syndromes could emerged as this: NS and NS-ML are mainly related with activators of the RAS/MAPK cascade (i.e., RAS or RAF activators), but NF and LS are associated to RAS inhibitors. In addition, CS and CFCS mutations hit the backbone of the pathway, while CS being centered on RAS and CFCS on downstream kinases (Atay and Skotheim, 2017).

##### Future prospective

With the continuous investigation and further understanding of causal mutations and functional analysis of pathophysiological consequences of RASopathies, tremendous advances have been made in the past 30 years. Whereas, given the current fragmentary view, the complexity of RASopathies determined that more issues and challenges lie ahead, such as unidentified causal genes in patients with RASopathies, further functional analyses of the newly discovered mutations, the precise mechanisms underlying the RASopathies (similarities and differences between RASopathies), and the variable expression of a gene mutation (Atay and Skotheim, 2017). In addition, we should take into account of the endocrine and metabolic prospective to interrogate the interactions and contributions of different mutations to the homeostasis imbalance and global phenotype. Moreover, additional factors, such as environmental, age-related and sex-related modifiers, may multifold the difficulty to decipher its pathophysiological process.

#### Neurological and Psychiatric Disorders

Alzheimer’s disease (AD) is the most common progressive neurodegenerative disorder, affecting more than 30 million people worldwide (Stornetta and Zhu, 2011). The disorder is characterized by early deficits in learning and memory followed by loss of other higher cognitive functions, which is correlated with synaptic depression and then neuronal degeneration (Haass and Selkoe, 2007). It is still unclear that what factors determined the age of onset and how the selective dysfunction of neurons in the brain been affected. A hallmark of AD pathology is the generation of amyloid beta (Aβ) from the amyloid precursor protein (APP) by APP-cleaving enzyme 1 (β-secretase, BACE1) (Schoneborn et al., 2018). Studies showed the underlying mechanism for physiological regulating BACE1 stability and activity in its GTP-bound state was Rheb GTPase, which induced mammalian target of rapamycin (mTOR) activity. Protein levels of BACE1 and Aβ generation are suppressed upon Rheb overexpression (Schoneborn et al., 2018). The interaction of GTP-activated Rheb with BACE1 stimulates its degradation via the proteasomal and lysosomal pathways (Shahani et al., 2014). Recently correlation study implied that the nutrient signaling might regulate cognitive functions in mammals by regulating Rheb–BACE1 and Rheb–mTOR pathways activity, which also orchestrated a potential new therapeutic target for Alzheimer’s-associated memory dysfunction (Shahani et al., 2017).

Parkinson’s disease (PD) is the second most common neurodegenerative disorder following Alzheimer’s disease, affecting 1–2% of the population above 60 years of age (Karimi-Moghadam et al., 2018). Although most PD cases occur sporadically, mutations in several genes, such as *SNCA* (α-synuclein), *PARK2* (parkin), *DJ-1*, *PINK1*, *ATP13A2*, *VPS35* (vacuolar protein sorting 35), *EIF4G1* (eukaryotic initiation factor 4G1) and *LRRK2* (leucine-rich repeat kinase 2), have been identified in hereditary PD (Tsika and Moore, 2013). LRRK2 is a large, multifunctional protein with a central catalytic GTPase/kinase core flanked by several protein-binding domains. Seven missense mutations, clustered in the Ras-of-complex (ROC) GTPase domain, C-terminal-of-ROC (COR) and kinase domains, segregate with PD in affected families (Atashrazm et al., 2018; Pfeffer, 2018). Dopaminergic signaling also plays a critical role in the pathogenesis of PD, and dopamine transporter (DAT) serves as a primary mechanism for terminating dopaminergic signaling (Shi et al., 2013). Rit2 (Rin) was recently identified as a protein that interacts with the DAT C-terminal endocytic domain, implying a role of Rin signaling in the regulation of DAT trafficking (Navaroli et al., 2011). In addition, studies from the perspective of immunity have also indicated that Rit2 polymorphisms affect the innate immune system, which in turn is responsible for some PD symptoms (Chan et al., 2016). Recent genome wide association study (GWAS) and meta-analysis results introduced Rit2 as a novel susceptibility locus, in association with decreased Rin expression in the substantia nigra pars compacta (SNc) of PD patients (Bossers et al., 2009; Latourelle et al., 2012; Pankratz et al., 2012). However, the results of RIT2 polymorphisms studies in different populations are controversial, which may be due to genetic context difference and environmental factors (Daneshmandpour et al., 2018). Therefore, further functional study, animal study and larger study with various population samples might give more detailed role of RIT2 in cells and groups in PD pathogenesis.

Huntington’s disease (HD) is a fatal autosomal-dominant neurodegenerative disease caused by CAG repeat expansion in exon 1 of *huntingtin*, which encodes the protein huntingtin (Htt). HD results in early loss of medium spiny neurons in the striatum, which impairs motor and cognitive functions (MacDonald et al., 1993). In HD, Htt contains an expanded poly-glutamine (poly-Q) tract. Under healthy conditions, Htt promotes signaling through mTORC1 (mammalian target of rapamycin complex 1). In the case of HD, the poly-Q tract potentiates the signaling by promoting the formation of a ternary complex of Htt-Rheb-mTOR, leading to enhanced mTORC1 activity (Sathasivam et al., 2013). In the striatum, Rhes (Ras homolog enriched in the striatum) serves as a key activator of mTORC1 (Pryor et al., 2014). Knockout of Rhes reduces mTORC1 activity and attenuates the adverse responses of L-DOPA-induced dyskinesia (Subramaniam et al., 2011; Lee et al., 2015). In addition, Rhes facilitates SUMOylation, a process implicated in HD pathogenesis (Subramaniam et al., 2009). Because Rhes is highly expressed in the striatum (Spano et al., 2004), it has been proposed that Rhes-Mutant Htt interactions may underlie the prominent striatal degeneration observed in HD. There is evidence that impaired Rhes/mTORC1 activity is relevant to the notable striatal pathogenesis in HD, which suggests that impaired mTORC1 function may represent a fundamental mechanism underlying the complex disease phenotypes of HD (Lee et al., 2015).

Autism spectrum disorders (ASD) are a continuum of complex neurological disorders interfering with normal social behavior and cognitive development (Hamedani et al., 2017). They are common neurodevelopmental diseases with onset prior to the age of three, affecting almost 1% of individuals worldwide (Liu et al., 2016). Children with ASD often show a large head circumference and develop epilepsy, and nearly half display severe intellectual disability (Stornetta and Zhu, 2011). ASD has a strong but complex genetic component. A recent study identified an increase in RIT1 transcript levels in patients with autism, implying that Rit may represent an ASD susceptibility gene (Garbett et al., 2008). Considering that several factors, such as abnormal assembly of synapses and dendritic spines, contribute to autism’s pathogenesis, the Rit signal pathway that regulates synaptic development and function might be a novel potential therapeutic target (Ebert and Greenberg, 2013).

Schizophrenia is a devastating psychiatric disorder characterized by reality distortion, with onset in late adolescence and unclear etiology (Glessner et al., 2010). Common features are positive symptoms, such as hallucinations, delusions, disorganized speech, and negative symptoms, such as social deficits, lack of motivation, anhedonia, and impaired emotion processing, as well as cognitive deficits with occupational dysfunction (Arajarvi et al., 2005). Previous studies have reported various copy number variations (CNVs) related to schizophrenia (Piluso et al., 2017; Zhuo et al., 2017). Several recent studies have suggested that the *rit2* gene might be involved in the pathogenesis of schizophrenia (Glessner et al., 2010). When compared with control subjects, patients with schizophrenia present a significant enrichment of common interstitial deletions (Bouquillon et al., 2011). Among the deleted genes, *rit2* was identified as a candidate gene for language delay, mental retardation, and behavioral abnormalities (Bouquillon et al., 2011). Evidence indicates that *ret* and *rit2*, both Ras-related genes important for neural crest development, are significantly affected by CNVs (Glessner et al., 2010). Rit2 is involved in the ubiquitin E3 ligase growth factor pathway that affects mitochondrial dysfunction, which is linked with both schizophrenia and autism (Prabakaran et al., 2004).

The protein interactions in the cells are common. Malfunction or alterations in the protein-protein interaction may influence vast biological functions. Selected members of Ras subfamily and their related possible interacting proteins are listed in the Table 2. Specifically for RIT2, evidence suggests that proteins interacting with RIT2 may cause or relate to similar clinical signs or diseases, which implied that RIT2 might be a potential candidate gene underlying several neurological diseases, such as PD, schizophrenia and autism (Daneshmandpour et al., 2018).

Considering the new insight of the role of members of Ras subfamily in neuro- psychiatric disorders and metabolic regulation, substantial novel therapies might be developed or repurposed based on the extensive studies of Ras subfamily signaling in the context of cancer. For example, evidence suggests trametinib, a small molecule inhibitor targets the MEK kinase with high specificity, extends lifespan in *Drosophila* and protects against the malfunction of genetic induced obesity in mice (Slack et al., 2015).

## The Rho Subfamily in Non-Neoplastic Cerebral Diseases

In the mid-1980s, Madaule and Axel (1985) serendipitously identified the first Ras homolog in *Aplysia* sea slugs, and named it Rho. In recent decades, the Rho subfamily of small GTPases has been shown to regulate many aspects of basic cellular processes such as cell polarity, cell movement, cell–cell interaction, cell proliferation and differentiation, cell morphology, secretion, adhesion, gene expression and survival (Watabe-Uchida et al., 2006; Boureux et al., 2007; Cingolani and Goda, 2008; Peru Y Colón de Portugal et al., 2012; Goitre et al., 2014). More than 20 Rho members, which are structured into six subclasses, are found in all eukaryotic cells as a molecular switch for actin cytoskeleton reorganization (Hall, 2012; Narumiya and Thumkeo, 2018). The Rho subfamily of small GTPases plays essential roles from changes in intracellular cytoskeleton dynamics to extracellular message exchange (Zamboni et al., 2018). In particular, Rho GTPases regulate dendritic arborization, spine morphogenesis, growth cone development, and axon guidance (Stankiewicz and Linseman, 2014). The Rho subclass, including RhoA, RhoB, and RhoC, predominantly promotes the formation of actin stress fibers and focal adhesion (Aspenstrom et al., 2004; Fernandez-Sauze et al., 2009). The Rac subclass (Rac1, Rac2, Rac3, and RhoG) promotes actin chain formation during the branching of lamellipodia, and the cell division cycle. 42 GTP-binding protein (Cdc42) subclass (Cdc42, TC10/RhoQ and TCL/RhoJ) mainly controls the formation of actin microspikes and filopodia (Azzarelli et al., 2014; Goitre et al., 2014).

Rho signaling is involved in several cerebral diseases, including intellectual disability (ID), epilepsy, drug addiction, HD, amyotrophic lateral sclerosis (ALS), and AD, and it acts by regulating axonogenesis, neuronal migration and synaptic plasticity (Table 3) (Mendoza-Naranjo et al., 2007; Locke et al., 2009; Zhang Y. et al., 2015; Li et al., 2016; Wang et al., 2017; Zimering, 2018; Costain et al., 2019). Rac1 and Rac3 are associated with intelligence by regulating key cellular functions in the central nervous system (Reijnders et al., 2017; Costain et al., 2019). Moreover, it was confirmed that missense mutations in Rac3 cause severe intellectual disability and brain malformations in humans (Costain et al., 2019). Previous studies have shown that X-linked ID is related to mutations in genes that code for regulators of the small-GTPase family such as Rac/Cdc42 guanine nucleotide exchange factor 6 (αPIX), RhoGEF and trio Rho guanine nucleotide exchange factor (TRIO) (Lelieveld et al., 2016; Reijnders et al., 2017; Zamboni et al., 2018).

Rac1 is essential for diverse forms of learning, and contributes to extinction of an established memory during drug withdrawal or alcohol use disorders (Peru Y Colón de Portugal et al., 2012; Wang et al., 2017). A recent study provided evidence that Rac1-dependent GABA-A receptor endocytosis plays a crucial role in the extinction of aversive memories (Wang et al., 2017). Furthermore, Rho signaling is involved in regulating neuronal synaptic plasticity in the nucleus accumbens (NAc) and ventral tegmental area (VTA), which both play crucial roles in the reward circuitry (Deguchi et al., 2016). These actions of Rho-associated kinase (ROCK) signaling might also be related to synaptic plasticity in the lateral amygdala and prelimbic prefrontal cortex, which were also identified as regulators in reward circuits (Lamprecht et al., 2002; Swanson et al., 2017). Furthermore, these studies have shown that Rac1 plays a role in drug addiction by regulating synaptic plasticity and the neuronal projection network. It has also been observed that cocaine reduces the phosphorylation of Ezrin/Radixin/Moesin proteins (ERM) in the NAc by downregulating RhoA-Rho kinase signaling, which may importantly contribute to the initiation of synaptic changes in this site, leading to drug addiction (Kim et al., 2009).

Cdc42 is a small GTPase of the Rho-subfamily that acts as a multifaceted key regulator of neuronal structure and function (Bustelo et al., 2007; Govek et al., 2011; Zhang Y. et al., 2015). Cdc42 plays an important role in regulating epileptic seizures (Zhang Y. et al., 2015). Cdc42 regulates the availability of presynaptic sites for CaV2.1 calcium channel incorporation (Chen et al., 2003; Frank et al., 2009), and presynaptic activation of Cdc42 can mimic the effects of electrical activity that promotes synaptic maturation and plasticity (Shen et al., 2006). Cdc42 is over-expressed in the human cortex of the temporal lobe and in the hippocampus of intractable epilepsy patients after an anterior temporal lobectomy (Xiao et al., 2007). Zhang Y. et al. (2015) demonstrated the effect of Cdc42 on the function of hippocampal CA1 pyramidal neurons and revealed that blocking Cdc42 decreases the spontaneous action potentials (APs), and increases both the miniature inhibitory postsynaptic current (mIPSC) and evoked inhibitory postsynaptic current (eIPSC) (Zhang Y. et al., 2015).

Although Rho subfamily has gained the most attention for its putative role in numerous neurodegenerative diseases, the precise mechanisms as a therapeutic target remain controversial and uncertain. In PD, Rho signaling pathway is a promising therapeutic target (Labandeira-Garcia et al., 2015). Some studies also suggest that mutations in LRRK2 can lead to a decrease in activation of Rac1 related Rho signaling, which causes disassembly of actin filaments leading to modulate cytoskeletal outgrowth and vesicular dynamics, including autophagy (Boon et al., 2014). These functions likely impact modulation of α-synuclein aggregation and associated toxicity in the pathophysiology of PD (Konno et al., 2012). A previous study revealed that ROCK inhibition protects against neuronal death induced by neurotoxins (Borrajo et al., 2014). ROCK regulation may provide a new neuroprotective strategy for neurodegenerative diseases, and it has to be settled urgently to develop more potent and selective ROCK regulators.

## The Rab Subfamily in Non-Neoplastic Cerebral Diseases

The Rab subfamily, which comprises more than 60 members, is currently the largest branch of the Ras superfamily of small GTPases (Wennerberg et al., 2005; Diekmann et al., 2011). This protein family is primarily related to various aspects of membrane and protein traffic in the endocytic and secretory pathways (Wennerberg et al., 2005; Cherfils and Zeghouf, 2013; Goitre et al., 2014; Gao et al., 2018). Therefore, dysregulation of Rab GTPases may lead to the pathogenesis of some diseases (Table 3). Many neurodegenerative diseases are characterized by dysfunction of membrane and protein traffic in neurons (Zhang X. et al., 2018). The Rab subfamily has also been related to neurodegenerative disorders such as Alzheimer’s disease and PD (Wilson et al., 2014; Saridaki et al., 2018; Yan et al., 2018). Previous studies have shown that Rab3A, Rab6, Rab8A/Rab8, Rab23, and Rab27b are highly expressed in the brain and participate in synaptic vesicle exocytosis, postsynaptic glutamate receptor dynamics, neurite growth and neural development (Takamori et al., 2006; Ng and Tang, 2008; Uno et al., 2016). Rab3 is the most abundant Rab protein in the brain. It is localized in synaptic vesicles and participates in their fusion and neurotransmitter release (Ng and Tang, 2008; Shin, 2014).

Alzheimer’s disease is clinically characterized by progressive cognitive impairment and memory loss (Bejanin et al., 2017). Two classical pathological features of AD are aberrant phosphorylated forms of tau protein and pathologically generated Aβ peptides (Lee et al., 2001; Alavi Naini and Soussi-Yanicostas, 2015; Bejanin et al., 2017; Bourdenx et al., 2017; Sun et al., 2018). Rab GTPases are implicated in multiple pathological mechanisms, including Aβ production and accumulation in AD (Li, 2011; Ridge et al., 2017; Rodriguez et al., 2017; Yan et al., 2018). Rab1B was found to play a key role in APP trafficking from the ER to the Golgi (Dugan et al., 1995). A study has shown that downregulation of Rab1B blocked APP transport in the ER/Golgi and significantly inhibited Aβ secretion (Dugan et al., 1995). Upregulation of either the early endosome protein Rab5 or the late endosome protein Rab7 increased Aβ trafficking from the cytoplasm to the lysosomes (Li et al., 2012). Previous post mortem studies have shown that the levels of Rab7A are elevated in the brains of AD model mice. Furthermore, some recent studies have shown that Rab7A may regulate tau secretion and tangle propagation in AD (Rodriguez et al., 2017; Zafar et al., 2017). Moreover, Rab11A-positive recycling vesicles accelerated cellular Aβ accumulation (Li et al., 2012).

A large amount of literature has reported the importance of the interaction between Rab GTPases and LRRK2 in PD (Steger et al., 2016; Alessi and Sammler, 2018; Eguchi et al., 2018; Jeong et al., 2018; Madero-Perez et al., 2018; Mir et al., 2018; Pfeffer, 2018). It has been reported that autosomal dominant missense mutations within the LRRK2 gene account for 1–2% of all cases of PD (Paisan-Ruiz et al., 2013). Similarly, variations at the LRRK2 locus also mildly increase the risk for idiopathic PD (Paisan-Ruiz et al., 2013; Domingo and Klein, 2018). A previous study verified that LRRK2 phosphorylates a subgroup of Rab GTPases which includes Rab7, Rab8A, and Rab10, and plays a crucial role in membrane and protein traffic (Gomez-Suaga et al., 2014; Roosen and Cookson, 2016; Steger et al., 2016, 2017). Consistently with the importance of LRRK2 for PD, Rab subfamily members can be seen as regulators of membrane trafficking. Furthermore, Rab phosphorylation was found to be altered *in vivo* in all the related pathogenic processes (Steger et al., 2016, 2017). Some reports showed that Rab35 and Rab39B may be implied in the pathogenesis of PD (Chiu et al., 2016; Lin et al., 2017). There is increasing evidence that mutations in Rab29 or Rab39B are related to the impairment of membrane trafficking relevant for PD (Ben-David and Tu, 2015; Domingo and Klein, 2018; Purlyte et al., 2018).

Hearing loss often results in plastic changes in the central auditory pathways, which are also related to members of the Rab family of small GTPase (Dong et al., 2010; Mulders et al., 2014). Gene expression of Rab3A and Rab3GAP1 fas found to be decreased in the paraflocculus after acoustic or mechanical cochlear trauma (Dong et al., 2010; Mulders et al., 2014). Moreover, early modulation of Rab GTPase gene expression in the paraflocculus may affect auditory processing by regulating the release of neurotransmitters (Mulders et al., 2014).

Cone-rod dystrophy (CRD) is an inherited retinal dystrophy that belongs to the group of pigmentary retinopathies. It is characterized by primary loss of cone photoreceptors and subsequent or simultaneous loss of rod photoreceptors (Hamel, 2007; Roosing et al., 2013). A previous study has shown that mutations in Rab28 are associated with autosomal-recessive cone-rod dystrophy (Roosing et al., 2013). Rab28 is located in the basal body and ciliary rootlet, where it plays a crucial role in ciliary transport. Other studies have shown that primary ciliogenesis is associated Rab3A, Rab6A, Rab8, and Rab11, which play an indirect role in rhodopsin transport from the photoreceptors’ inner to the outer segments through the connecting cilium (Moritz et al., 2001; Satoh et al., 2005; Knodler et al., 2010).

## The Arf Subfamily in Non-Neoplastic Cerebral Diseases

Arf GTPases are a subfamily of proteins in the Ras superfamily that were identified as cargo displacement factors (Ismail et al., 2011). The roles of Arf GTPases include protein trafficking, lipid metabolism and trafficking, as well as actin remodeling in eukaryotic cells via their regulated GTP cycle (D’souza-Schorey and Chavrier, 2006; Jackson and Bouvet, 2014). According to sequence homology, there are four main classes of Arf proteins in mammals, namely Class I (Arf1–3), Class II (Arf4–5), Class III (Arf6), and unclassified members (Rojas et al., 2012; Jackson and Bouvet, 2014). Arf1 is the first member of this small GTPase subfamily, which is viewed as key regulators of eukaryotic cell organization (Kahn and Gilman, 1986; Jackson and Bouvet, 2014). Mutations in Arf1 have been shown to be related to autosomal recessive periventricular heterotopia, a disorder that leads to severe malformation of the cerebral cortex (Sheen et al., 2004).

In the last two decades, a series of cerebral diseases associated with Arf GTPases have been studied (Table 3). Previous studies have shown that many Arf-regulated ER–Golgi trafficking processes are defective in ALS (Saxena et al., 2009; Wang et al., 2011; Zhai et al., 2015; Atkin et al., 2017). Moreover, a study has shown that regulation of Arf signaling reverses mutant protein toxicity in ALS by decreasing ER stress and stimulating various types of autophagy in cell lines and animal models (Zhai et al., 2015). Recent studies have shown that Arl proteins which belong to Arf GTPases are associated with retinitis pigmentosa (Veltel et al., 2008a; Zhang H. et al., 2015; Hanke-Gogokhia et al., 2016b). In the pathogenetic process of retinitis pigmentosa, Arl2/Arl3 signaling plays important roles in photoreceptor function by regulating lipid-modified membrane-associated proteins (Hanke-Gogokhia et al., 2016b). The formation of a ternary complex between Arl3, its cognate GAP RP2 and its retinal effector HRG4 is also of great importance for photoreceptor function (Veltel et al., 2008b). In a rodent model, regulating the activities of Arl3 GAP can reduce the severity of photoreceptor disease (Zhang H. et al., 2015).

## The Ran Subfamily and Unclassified Ras Superfamily Members in Non-Neoplastic Cerebral Diseases

Ran (Ras-related Nuclear protein) is generally encoded by a single ortholog in eukaryotes (Reiner and Lundquist, 2018). The classic function of Ran is to regulate the cycle of nuclear import and export (Reiner and Lundquist, 2018). There are few literature reports on the relationship between Ran and cerebral diseases (Table 3). A study indicated that the expression of Ran is reduced in AD, and Ran is a pivotal molecule in nucleocytoplasmic transport in AD pathophysiology (Mastroeni et al., 2013).

Ran also plays crucial roles in frontotemporal lobe degeneration (FTLD) by regulating TDP-43 induced retinal neurodegeneration (Ward et al., 2014). FTLD comprises a group of disorders, and is clinically characterized by behavioral and personality changes, language impairment, as well as deficits in executive functioning, and is pathologically associated with degeneration of frontal and temporal lobes (Morris et al., 2012; Pottier et al., 2016). The expression of Ran is found to be reduced by nuclear depletion of TDP-43 in a Grn-KO induced rodent model of retinal neuronal loss (Ward et al., 2014). Retinal neurodegeneration as a new phenotype involves the reciprocal loss of Ran in progranulin-deficient FTLD via an underlying mechanism related to nuclear TDP-43 (Ward et al., 2014). There are also some unclassified members in the Ras superfamily of small GTPases (Rojas et al., 2012), and these also play vital roles in cerebral diseases. Gispert et al. (2015) studied synucleinopathy-induced expression changes in the mouse brain and identified 49 midbrain/brainstem-specific transcriptional dysregulations, including Rabl2A downregulation. Mitochondrial Rho GTPase 1 (MIRO1), which is encoded by the *rhoT1* gene (Fransson et al., 2003; Tada et al., 2016; Lahiri and Klionsky, 2017), is involved in mitochondrial homeostasis and apoptosis, as well as PD and cancer (Anvret et al., 2012; Jiang et al., 2012; Schwarz, 2013; Sheng, 2014; Van Der Merwe et al., 2015; Lahiri and Klionsky, 2017). A recent study showed that MIRO1 is a potential adaptor for microtubule based peroxisome motility in mammalian cells (Castro et al., 2018). Another study has revealed that Rab20 is related to a genetic mechanism of longitudinal cognitive changes during the transition period from mild cognitive impairment to AD (Lee et al., 2017). Liang et al. (2012) found that Rab20 is substantially upregulated during the acute phase of brain inflammation, and also plays crucial roles in the subsequent inflammatory responses in the brain.

## Conclusion

Knowledge about the roles of the Ras superfamily of small GTPases in cerebral diseases has considerably grown in the past 30 years. Currently, many research teams all over the world are also trying to identify novel small GTPases, which can be viewed as new regulators and effectors that control the crucial structure and biological functions affected by cerebral diseases. In particular, these important small GTPases have improved the development and applications of cellular and animal disease models. In this review, we mainly discussed how Ras-superfamily GTPases contribute to a range of human cerebral diseases as specific effectors in a series of complex mechanisms. Finally, a helpful summary of all described small GTPases from the Ras superfamily that play a role in cerebral diseases is shown schematically in Tables 1–3. Therefore, seeking treatment solutions for the related diseases by identifying the as-yet unknown physiological or pathological functions of more than 167 Ras superfamily members will open new research directions and fields in the following decades.

## Author Contributions

X-LW and YW designed the study. LQ and CP collected, analyzed data, and wrote the manuscript. S-MH and BL interpreted the data and revised the manuscript. YW edited and polished the manuscript. G-DG and X-LW finalized the manuscript. All authors critically reviewed content and approved final version for publication.

## Conflict of Interest Statement

The authors declare that the research was conducted in the absence of any commercial or financial relationships that could be construed as a potential conflict of interest.

## Supplementary Material

Table S1 G-domin regions of 167 small GTPases found in humans.
